# Challenges in Infection Epidemiology: On the Underreporting of Norovirus Gastroenteritis Cases in Germany

**DOI:** 10.3390/ijerph17010314

**Published:** 2020-01-02

**Authors:** Felix Martin Hofmann, Edward Olawumi, Martina Michaelis, Friedrich Hofmann, Ulrich Stößel

**Affiliations:** 1 Research Centre for Occupational and Social Medicine (FFAS), 79098 Freiburg, Germany; olawumi@ffas.de (E.O.); michaelis@ffas.de (M.M.); hofmann@ffas.de (F.H.); stoessel@ffas.de (U.S.); 2 Institute of Earth and Environmental Sciences, University of Freiburg, 79104 Freiburg, Germany

**Keywords:** norovirus gastroenteritis, notifiable disease, epidemiology, secondary data, public health, underreporting

## Abstract

It is commonly accepted that the number of officially reported incidences of norovirus (NoV) according to the German Protection against Infection Act (*Infektionsschutzgesetz*) does not reflect the ‘true’ incidence of NoV in Germany. This study aims to reveal the reasons for the underreporting of NoV cases by comparing secondary data. Methods: NoV incidence (cases per 100,000 reference persons) in the age group 18–65 was derived from register data of four different sources in the German public health system (2011–2015): Statutory health insurance in the federal state of Lower Saxony (AOK; in- and outpatient cases), the Research Institute of Ambulatory Health Care in Germany (ZI; outpatient cases), the German Federal Statistical Office (inpatient cases; DESTATIS), and the Robert Koch Institute (RKI SurvStat; health reporting data). Results: the incidence derived from the AOK in Lower Saxony varied between 49 and 66 NoV cases per 100,000 persons and was thus lower than at the federal level. Incidences of all inpatient and outpatient data were lower than the incidence according to the RKI in the last 2–3 years of the observation period. Conclusions: the disagreement between NoV incidences calculated from secondary inpatient and outpatient data and the respective numbers published by the RKI can be regarded as an indication that not all NoV cases were reported to public health authorities. This might be due to missed cases during the notification procedure or misclassification of gastroenteritis cases by general practitioners. Considering the limitations associated with analyzing secondary data, the appropriateness of these assumptions should be verified in future studies.

## 1. Introduction 

Noroviruses (NoV) are responsible for the majority of non-bacterial gastrointestinal inflammation cases in children and adults and cause about 18% of acute gastroenteritis [1]. Outbreaks in hospitals, community facilities, and kitchens occur regularly and seasonally during winter months [2]. The highly infectious viruses are transmitted within a short period of time, usually by fecal-oral or oral ingestion of virus-containing droplets, which are excreted by vomiting and/or diarrhea [3]. In individual cases, a NoV infection can be fatal in older and immunodeficient people [4,5,6]. From an epidemiological point of view, NoV gastroenteritis was the second most common infectious disease after influenza in 2017, with 89 incidences of infection per 100,000 inhabitants according to the reporting data of the Robert Koch Institute (RKI) [7].

In view of the rapid progression of the disease, varying degrees of symptom tolerance among patients, and presumably different diagnostic efforts by physicians, a high incidence of unreported cases can be assumed [8,9,10]. International comparisons are therefore difficult to make in view of different monitoring procedures and reporting channels. Another difficulty in estimating the ‘true’ NoV incidence in Germany stems from changes to the German Protection against Infection Act from 2011. Since then, only ‘clinically laboratory-confirmed’ cases and no longer all cases, including symptomatic cases recorded ‘clinically-epidemiologically’, have to be reported to the RKI. This change was felt necessary, as ‘clinical-epidemiological’ cases are recorded by the public health authorities and not by the laboratories. These cases accounted for half of the officially notified NoV cases in the 2006/2007 and 2007/2008 seasons. Due to the disproportionally high workload for public health authorities and the lack of a suitable software for the notification of ‘clinical-epidemiological’ NoV cases at that time, the reporting system was changed to reduce the workload for public health authorities [11]. The case definition for the Dengue virus was also modified in 2011 and the increasingly used NS1 antigen test has been accepted as evidence for the detection of Dengue virus infections [11]. Further changes in other case definitions (brucellosis, giardiasis, listeriosis etc.) were published in 2014 [12]. After the modification of the NoV case definition in 2011, the German reporting system became, similar to that in the United Kingdom and Sweden where the respective reporting systems rely on laboratory evidence [13,14]. Clinically laboratory-confirmed NoV cases generally represent only a fraction of all NoV cases [15], which also makes time series analyses more difficult. Underreporting of NoV infections can also be considered a challenge of policy making, as the costs of NoV outbreaks for health systems and the economic damage remain largely unknown. The analysis of the economic impact of NoV outbreaks is mainly restricted to case studies [16,17].

One aim of our method-mix study *NoroEpi* [18] was to approach the ‘true’ incidence of NoV from an epidemiological point of view. In this project, we further aimed at evaluating the significance of NoV in occupational health. We also interviewed occupational health [19] and hygiene [20] specialists to get insights in reporting and laboratory testing routines. In this article, we describe the treatment indications of the diagnosis A08.1 ‘Acute gastroenteritis caused by Norovirus (Norwalk virus)’ of the International Classification of Diseases (ICD10) in various secondary data (so-called routine data). For that purpose, we exemplarily analyzed the data of one statutory health insurance in a selected federal state, as well as data from health service providers (hospitals and general practitioners). In a second step, the results were compared with respective reporting data from the Robert Koch Institute according to the German Infection Protection Act (*Infektionsschutzgesetz*; IfSG) to identify discrepancies and draw conclusions about the reasons for the underreporting of NoV cases.

## 2. Methods

For the comparative evaluation of routine data statistics, we largely followed the recommendations for the consideration of consensus reporting standards in the analysis of secondary data, as developed by Swart et al. [21]. Our analyses included data for the years 2011 to 2015, because they were available from all four of the following data sources. These are described here in more detail:

1. Billing information data of persons aged 18 to 65 insured through the statutory health insurance ‘*Allgemeine Ortskrankenkasse*’ in the federal state of Lower Saxony (AOK) whose outpatient or inpatient treatment was coded as ICD10 number A08.1. As the demographic structure (age and gender) of the population insured through the AOK hardly differed from the demographic structure of the population of Lower Saxony as a whole [22], the calculated incidences could be compared with data from the following three sources.
2. Billing information data from the Central Institute for Statutory Health Insurance Physician Care (*Zentralinstitut für die Kassenärztliche Versorgung*; ZI) for all outpatients treated in Germany with statutory health insurance given the diagnosis A08.1. The data for the age group 20–64 years were analyzed to achieve approximate comparability with the incidence of outpatient diagnosis A08.1 reported in the billing data from the AOK.
3. Treatment data on the diagnoses of hospital patients according to the four-digit code of the International Classification of Diseases (ICD, version 10) A08.1 [23,24] kept at the German Federal Statistical Office (*Deutsches Statistisches Bundesamt*; DESTATIS). According to the definition of the Federal Statistical Office given in the datasets [23,24], hospital cases with several flat-rate case diagnoses were subsumed under the ICD diagnosis that was primarily responsible for inpatient treatment and was considered the discharge diagnosis. For this reason, nosocomial cases are not explicitly reported in the case figures of the Federal Statistical Office. Here, too, the data of 20–64-year-olds were aggregated for comparison purposes.
4. NoV reporting data from the Robert Koch Institute according to the reference definition ‘clinical laboratory diagnostic’ for Lower Saxony, as well as the entire federal territory of Germany from the Internet portal SurvStat@RKI 2.0 [7].

All incidence calculations (NoV patients per 100,000 insured persons or inhabitants) were based on case numbers, even if. In a few exceptions, a patient may have fallen ill several times during the observation period. The limitation to the age range of 18–65-year-olds and 20–64-year-olds was also made against the background of preparing the data for further occupational epidemiological analyses [25]. Accordingly, the following presentation of the results concerns only this age group.

## 3. Results

### 3.1. NoV Diagnoses in the Routine Data of the AOK in Lower Saxony

**Figure 1:**
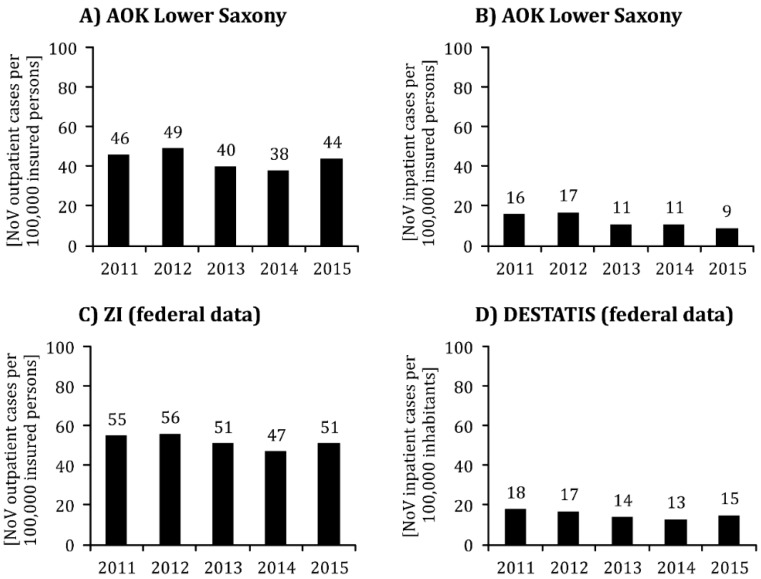
Incidence (cases per 100,000 insured persons or inhabitants) of the ICD10 diagnosis A08.1 ‘Acute gastroenteritis caused by Norovirus (Norwalk virus)’ from 2011 to 2015. Legend: (**A**) only outpatient cases among 18–65 year old insured persons of the statutory health insurance (*Allgemeine Ortskrankenkasse*; AOK) in Lower Saxony; (**B**) only inpatient cases such as A); (**C**) only inpatient cases among 20-64 year old insured persons of the statutory health insurance funds in Germany as a whole according to data of the Central Institute for Statutory Health Insurance Physician Care (*Zentralinstitut für die Kassenärztliche Versorgung*; ZI); (**D**) only inpatient cases in 20–64-year-old hospital patients in Germany as a whole (only main diagnosis) according to data from the German Federal Statistical Office (DESTATIS).

The number of insured persons of working age whose *outpatient* treatment was invoiced with the ICD10 diagnosis A08.1 was between 38 and 49 per 100,000 persons in the observation period (2011–2015). The NoV incidence was highest in 2012; while it stabilized in the following two years with significantly lower values (see Figure 1A).

**Figure 2:**
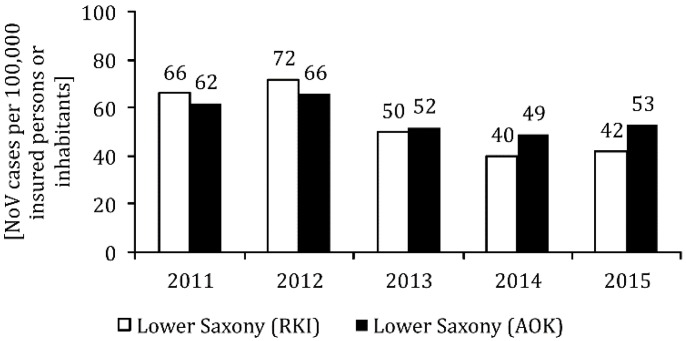
Noroviruses (NoV) incidence among 20 to 64-year-olds (cases per 100,000 inhabitants) in the years 2011 to 2015 in Lower Saxony according to the reference definition ‘clinical laboratory diagnostic’ of the Robert Koch Institute (RKI), as well as inpatient and outpatient NoV cases among the 18–64-year-olds insured at the AOK in Lower Saxony.

As expected, the number of inpatient NoV cases per 100,000 AOK insured persons was well below the incidence for outpatient cases (9 to 17 cases per 100,000 in the observation period). It also remained stably lower after 2012 than in the two previous years (see Figure 1B). The total number of outpatient and inpatient cases from 2011–2015 was between 49 and 66, with the peak already visible in 2012. The numbers stabilized at a lower level during the last three years of observation (62/66/52/49/53 cases per 100,000 insured persons in the course of the year; see also Figure 2).

### 3.2. Accounting Data of the Central Institute for Statutory Health Insurance Physician Care (ZI)

According to the nationwide data of the ZI, the incidence of *outpatient* ICD10 diagnosis A08.1, with 47 to 56 NoV cases per 100,000 persons in the observation period, also fluctuated at a higher level than the accounting data of the AOK in Lower Saxony. The ZI data also show a decline in the incidence of NoV after 2012 (see Figure 1C).

### 3.3. Hospital Billing Information Data of the German Federal Statistical Office (DESTATIS)

Similar to the AOK data (see Figure 1B), the data from the Federal Statistical Office shows only minor fluctuations during the observation period, with 13 to 18 inpatient NoV cases per 100,000 inhabitants (see Figure 1D). Here, too, the figures are comparatively lower for diagnoses after 2012 and are slightly above the NoV incidence in the group of inpatients with AOK insurance in Lower Saxony.

### 3.4. Reporting Data of the Robert Koch-Institute

**Figure 3:**
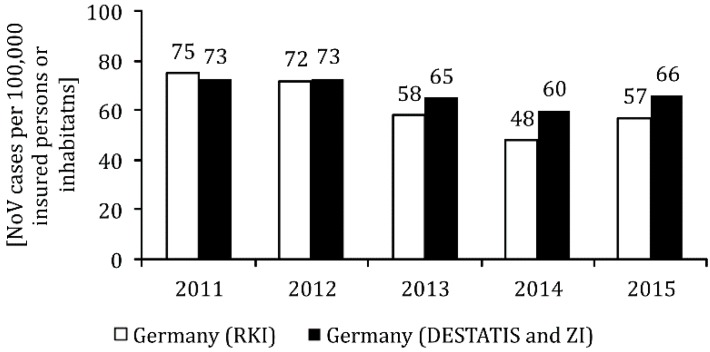
NoV incidence among 20 to 64 year olds (cases per 100,000 inhabitants) in the years 2011 to 2015 in Lower Saxony according to the reference definition ‘clinical laboratory diagnostic’ of the Robert Koch Institute (RKI) [7], as well as inpatient and outpatient NoV cases in the same age group in Germany as a whole according to data from the Central Institute for the Association of Statutory Health Insurance Physicians (ZI) and the Federal Statistical Office (DESTATIS).

Nationwide, the NoV case numbers per 100,000 inhabitants reported to the RKI according to the reference definition ‘clinical laboratory diagnostic’ were between 48 and 75 cases in the observation period (see Figure 2, bright bars). In Lower Saxony, the NoV incidence fluctuated between 40 and 72 cases (see Figure 3 bright bars). The temporal course of the reported NoV cases in Lower Saxony basically corresponds to the reporting data for Germany as a whole (see Figure 2, bright bars). However, with the exception of 2012, the incidence of reports from Lower Saxony was below the nationwide NoV incidence.

### 3.5. Reporting Data of the Robert Koch-Institute

The comparison between the NoV incidence in Lower Saxony published by the RKI (see Figure 2, light bars) and the outpatient and inpatient cases for the diagnosis A08.1 among those insured by the AOK Lower Saxony (see Figure 2, dark bars) shows that in the first two years of observation, the NoV incidence was lower in the data from the AOK Lower Saxony than the NoV incidence for Lower Saxony reported by the RKI. In 2013, the incidence of NoV from the two data sources was about the same, while in 2014 and 2015, the incidence of NoV among those insured by the AOK was 49 to 40 and 53 to 42 NoV cases per 100,000 persons, respectively. This was significantly higher than the NoV incidence in Lower Saxony reported by the RKI (Figure 2).

Taking the incidence of NoV from the DESTATIS and ZI data together (Figure 3, dark bars) and comparing it with the incidence of NoV for Germany as a whole as published by the RKI (Figure 3, light bars), the numbers generally agree during the first years after legislation was changed (2011 and 2012 respectively), with 73 to 75 and 73 to 72 NoV cases per 100,000 inhabitants, respectively. In the last three years of observation, the combined NoV incidence from the DESTATIS and ZI data was significantly higher than the overall NoV incidence in Germany published by the RKI. In 2014, the difference even amounted to 12 NoV cases per 100,000 persons.

## 4. Discussion

Our comparison of NoV incidences between 2011 and 2015 from four sources of routine data shows a fundamental agreement between the development of medically diagnosed and laboratory-confirmed NoV cases over time: after a peak in 2012, the values fell to a comparatively lower level. In contrast to the federal data from ZI and DESTATIS, it seems unlikely that the number of outpatient and inpatient NoV cases diagnosed per 100,000 insured persons of the AOK in Lower Saxony (western Germany) in 2012 is related to increased media attention as a result of the largest outbreak in eastern Germany to date in autumn 2012, in which around 11,000 people fell ill with NoV gastroenteritis as a result of eating contaminated frozen strawberries from China [26]. In fact, between early October 2012 and late December 2012, fewer cases were reported to the Lower Saxony State Health Office than in the comparable period of the previous year, which is why the peak of the NoV incidence is likely to be related to higher case numbers during the 2011/2012 NoV season [27]. Thus, the large NoV outbreak in autumn 2012 is only related to the peak NoV incidence at the federal level.

There are three possible explanations for the discrepancy between the slightly higher NoV incidence in the data from persons insured by the AOK and the reporting data of the RKI data for Lower Saxony for 2014 and 2015, and for the difference between the NoV incidence from the ZI/DESTATIS data and the RKI data published for the years 2013 to 2015:

1. It is possible that the hospital stay of a patient was billed with the main diagnosis A08.1, although the outpatient diagnosis A08.1 had already been made for this patient in advance. Thus, this patient was included in the ZI and DESTATIS data as both an outpatient and an inpatient case, whereas it was not entered more than once in the RKI database. This effect, with an artificial increase in NoV incidence, must also be taken into account when interpreting the insured person data of the AOK. On the other hand, the DESTATIS data for the years 2011 to 2015 did not include any nosocomial NoV cases, which is why the actual number of NoV cases in German hospitals may have been higher than suggested by the DESTATIS data. To what extent these contradictory effects have led to a reduction in data quality that cannot be answered here. Irrespective of this, it can be stated that different case definitions or a lack of harmonization of the secondary data we are looking to represent a limitation of our study. A further limitation is that the ZI/DESTATIS data is only available nationwide and a direct comparison between these and AOK data of a federal state is not possible. As can already be seen in the RKI statistics, reporting compliance in the eastern German states is higher than in most western German states, and thus influences the incidence rates [15].
2. The discrepancy may indicate that NoV cases were not recorded in the register of residents of the RKI during the last two years of observation among patients treated by SHI physicians, which would constitute a violation of the reporting obligation under §7(1) No. 36 IfSG. To what extent this effect plays a role cannot be clarified without further studies.
3. Furthermore, insufficient coding quality on the part of general practitioners (GP) is possible [10]. It is conceivable, for example, that the treatment of possible NoV cases was invoiced with the ICD10 diagnosis A08.1, although no reliable laboratory evidence was available. According to §7(1) No. 36 IfSG, laboratories must notify the responsible health authority of every person for whom an NoV-positive stool sample is available by patient name [3]. This notification is then forwarded by the health authorities to the competent state authority, which finally reports these figures to the RKI [8]. According to personal reports from individual representatives of the Public Health Service (7.2. and 19.2.2019), however, NoV cases are not reported by the laboratories only in exceptional cases. Accordingly, the NoV case numbers reported by the laboratories should be regarded as resilient.

On the basis of these considerations, the following explanations for the under-recording of NoV cases appear most likely:

1. a subjectively different experience of NoV symptoms may lead affected persons, whose infections are not confirmed in the context of an outbreak, to seek help from a doctor to varying degrees or (partly contrary to the official recommendations) stay away from work for varying lengths of time [10];
2. a varying diagnostic behavior of doctors, on the other hand, and thus a varying degree of willingness to arrange for an appropriate laboratory test if relevant symptoms are present. If physicians estimate the burden of NoV patients as low due to the temporary character of the disease, whereby a larger number of potential NoV cases may then be accounted for with the ICD10 diagnosis A08.4 ‘Virus-induced intestinal infection, not specified in more detail’ [10];
3. a varying capacity of local health authorities to press for laboratory diagnostic evidence to clarify the causative agent of infection in the event of outbreaks of gastroenteritis in community establishments [10]. In this context, we have unpublished data on gastroenteritis outbreaks in the catchment area of two health authorities. With the exception of outbreaks in hospitals and nursing homes for the elderly, insufficient laboratory diagnostic evidence was available from community institutions, especially day-care centers. However, as these are only two case studies, this trend cannot be generalized.

Due to e.g., economic constraints, it can be assumed that NoV infected employees in food processing companies do not always adhere to activity prohibitions and that sick personnel in hospitals do not always adhere to the 48 h [28] absence from work recommended by the RKI. In connection with the first assumption, it should be mentioned that the disregard of an activity prohibition according to § 42 IfSG by a trainee in a canteen probably led to 52 cafeteria visitors to suffer from NoV gastroenteritis [29]. Whether this is only an isolated case or whether employees in food processing companies regularly infiltrate bans on activities would have to be investigated by means of a targeted survey of personnel. A survey among nursing staff we conducted did not confirm the suspicion that employees in hospitals continued to work on a larger scale during an NoV infection [30]. On the other hand, the question arises as to how quickly NoV patients visit a doctor. In order to answer this question, it is advisable to conduct targeted quantitative interviews with NoV patients to find out which factors, including the severity of the disease, actually lead people to seek help from a doctor.

In connection with the use of the ICD10 ‘Contingency diagnosis’ A08.4, it should be noted that this outpatient diagnosis figure was invoiced 28 times more frequently on average than the figure A08.1 (standard deviation of 3.7) for insured persons of the AOK during the observation period. For this reason, it seems justified to assume that a considerable number of potential NoV cases may be hidden among the gastroenteritis cases accounted for in A08.4. A more precise proportion could be estimated by an epidemiologically based survey of general practitioners on their diagnostic and billing behavior. The results of a longitudinal study from Switzerland show that GPs do not routinely obtain laboratory diagnostic evidence in cases of gastroenteritis [31]. Experiences within this study show, however, that such projects are not easy to conduct.

According to the definition of the A08.1 ‘Acute gastroenteritis caused by Norovirus (Norwalk virus)’ diagnosis, the treatment of a patient can only be billed with this diagnosis if the NoV infection is proved by laboratory evidence. Due to the high sensitivity of reverse transcription-polymerase chain reaction (RT-PCR), noroviruses may also be detected in specimens from persons who are asymptomatically infected or have recovered from an earlier NoV infection, thereby leading to false-positive cases [2]. Hence, it may be the case that some persons whose diagnosis was billed with the A08.1 diagnosis were false-positive. This can be regarded as another shortcoming of the analyzed secondary data.

## 5. Conclusions

Overall, we show that the analysis of secondary data allows only limited conclusions with regard to the underreporting of NoV cases. Different case definitions and a lack of reconciliation of secondary data to avoid double-counted NoV cases are the main methodological limitations. These prevent clear statements from being made regarding the under-recording of NoV cases. However, targeted case studies can only partially solve this problem, since the individual case character of such studies must always be taken into account when interpreting the data obtained. Thus, there is still a considerable need for research in the development of a suitable methodology for testing previous hypotheses for the under-recording of NoV cases.
